# Novel Therapeutic Targets for Hypoxia-Related Cardiovascular Diseases: The Role of HIF-1

**DOI:** 10.3389/fphys.2020.00774

**Published:** 2020-07-15

**Authors:** Minxuan Liu, Gina Galli, Yilin Wang, Qiru Fan, Zhenzhong Wang, Xin Wang, Wei Xiao

**Affiliations:** ^1^State Key Laboratory of New-tech for Chinese Medicine Pharmaceutical Process, Lianyungang, China; ^2^Faculty of Life Sciences, The University of Manchester, Manchester, United Kingdom; ^3^Pharmaceutical Sciences, University of Maryland, Baltimore, Baltimore, MD, United States; ^4^Life Science and Technology, China Pharmaceutical University, Nanjing, China

**Keywords:** hypoxia, cardiovascular disease, therapeutic target, HIF, myocardial infarction, atherosclerosis

## Abstract

Insufficient oxygen availability (hypoxia) is a precursor to numerous cardiovascular diseases, including atherosclerosis, pulmonary hypertension, and heart failure. The main site of hypoxic injury in the human body is the mitochondria, where oxygen acts as the final electron acceptor in the process of oxidative phosphorylation. Hypoxia-inducible factor (HIF) is activated in hypoxic conditions and acts as an important modulator of diverse target genes in the human body. The downstream genes of HIF include vital modulators of cardiovascular-related signaling pathways. Therefore, it is hypothesized that HIF represents a potential therapeutic target for the treatment and prevention of cardiovascular diseases. In this short review, we introduce the pathophysiology of hypoxic injury in cardiovascular disease, and we conclude from convincing evidence that HIF can modulate relevant cardioprotective signaling pathways.

## Introduction

Oxygen is essential for most eukaryotic organisms to maintain normal cellular function and survival. As an important prerequisite for aerobic respiration, oxygen helps to generate ATP which provides the energy for organisms to maintain cellular homeostasis (Waypa et al., 2016). Mitochondria are the site of aerobic respiration and the largest consumer of cellular oxygen; they produce ATP via the tricarboxylic acid (TCA) cycle and the process of oxidative phosphorylation (OXPHOS) (Kim et al., 2006). However, when oxygen supply does not meet cellular ATP demand, termed hypoxia, cells are forced to employ anaerobic respiration which produces less than 1/10th of the aerobic ATP supply. Therefore, hypoxia is usually synonymous with cellular dysfunction and it may lead to cell death under chronic circumstances (Sendoel and Hengartner, 2014). It is well-known that this condition is a major cause of several severe cardiovascular diseases (Han et al., 2014).

In response to hypoxia and its direct impact on cell metabolism, the cell induces the expression of a large number of genes. In particular, hypoxia-inducible factor 1 (HIF-1) is a transcriptional heterodimer composed of an α-subunit (HIF-1α) and a β-subunit (HIF-1β) (Sousa Fialho et al., 2019). In normoxic conditions, the HIF-1α subunit is generated in the cytosol and degraded in an oxygen-dependent manner (Salceda, 1997). However, in hypoxic conditions, the degradation process is suppressed and HIF-1α is transferred into the nucleus to form a heterodimer with the β-subunit (Benson Ham and Raju, 2017). It is well-known that HIF-1 acts as a regulator of hundreds of target genes that can initiate distinct responses to low oxygen availability. While these regulated gene signaling pathways are known to mediate protective pathways in the short term, they can eventually lead to cardiovascular dysfunction (Abe et al., 2017). In this short review, we highlight the importance of HIF-1 cellular hypoxia and the pathophysiology of cardiovascular disease, and we offer insight into potential clinical therapies involving the modulation of HIF-1 pathways. Before we turn to the role of HIF-1 in cardiovascular disease, it is instructive to review the sequence of events leading to cellular dysfunction during hypoxia.

## The Cellular Response to Hypoxia

Under normoxic conditions, eukaryotes adopt aerobic respiration. In brief, glucose is broken down into pyruvate via glycolysis which yields 2 molecules of ATP. Pyruvate is then transported to the mitochondrial matrix and dehydrogenated by pyruvate dehydrogenase (PDH). The resulting product, acetyl coenzyme A, feeds into the citric acid cycle and is broken down into NADH and FADH_2_, which are the electron providers for OXPHOS. In the mitochondrial inner membrane, the electron transport chain (ETC) is the main stage for OXPHOS (Supinski et al., 2019). Electrons are transported from NADH and FADH_2_ to complex I (NADH CoQ reductase) and complex II (succinate CoQ reductase), respectively, where they are shuttled to complex III (ubiquinol cytochrome c reductase) by diffusible ubiquinone CoQ, and finally to complex IV (cytochrome c oxidase) by diffusible cytochrome c (Fuhrmann and Brune, 2017). Here, oxygen contributes to energy production by acting as the terminal electron acceptor. The electron transportation through the inner membrane provides the energy for complexes I, III, and IV to pump protons against their electrochemical gradient and create a proton motive force between the mitochondrial matrix and intermembrane space; this gradient provides the energy for complex V (ATP synthase) to convert ADP to ATP (Ham and Raju, 2017).

Under hypoxic conditions, mitochondrial function is severely impacted because the terminal electron acceptor, oxygen, becomes limiting. The lack of oxygen inhibits electron transport and OXPHOS, which reduces ATP production. In order to survive hypoxia, cells must adapt to the hypoxic condition and remodel aspects of the ETC. For example, the original ETC can be altered into supercomplexes, formed by complexes I, III, and IV. The remodeled supercomplexes maintain normal transportation of electrons, and they also help to inhibit the overproduction of reactive oxygen species (ROS) (Chaban et al., 2014). Under normal conditions, mitochondria generate ROS species, including superoxide and H_2_O_2_, from the leakage of electrons from the ETC; this process contributes to cellular signaling and does not cause damage to the cell (McElroy and Chandel, 2017). However, in hypoxic conditions, the lack of oxygen causes more electron slip, and ROS accumulates, leading to serious oxidative damage to numerous cellular components. The accumulation of superoxide radicals has a direct impact on the mitochondrial membrane; membrane permeability is increased and membrane potential is decreased. This instability of the mitochondrial membrane causes the leakage of cytochrome c and apoptotic protease activating factor into the cytosol (Ham and Raju, 2017). Therefore, the overproduction of superoxide radicals eventually causes cell death via apoptosis.

**FIGURE 1:**
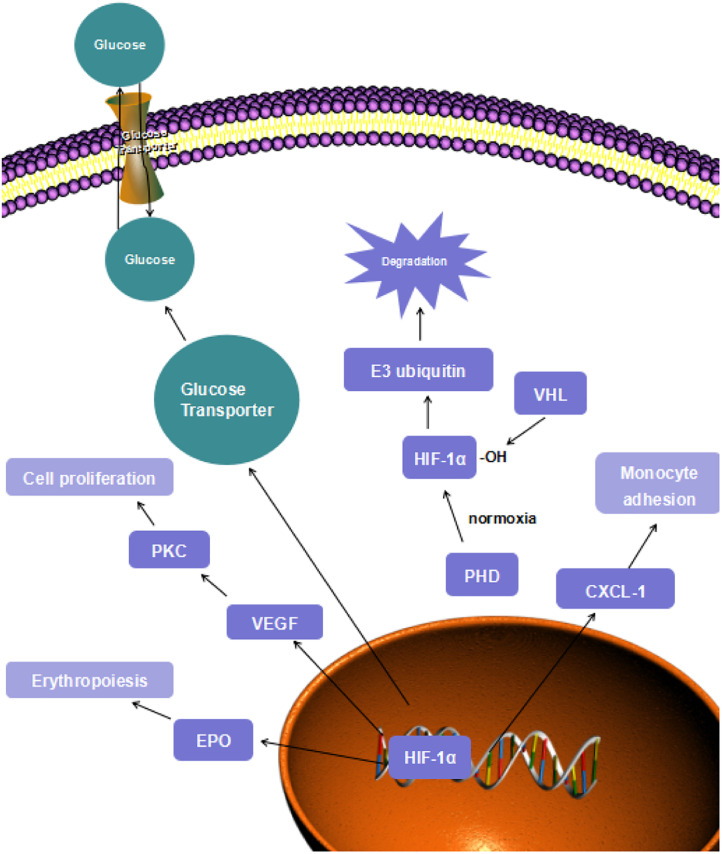
HIF-1α signaling.

Cardioprotective conditioning is a series of mechanisms involving various signaling pathways. Once ischemia occurs, conditioning progress will be induced to reduce damage to the cardiovascular system. An ischemia-induced myocardial stretch can be a physical stimulation to cardioprotection conditioning. As shown in Figure 1, the signaling pathway of protein kinase C (PKC) and ATP-dependent potassium (KATP) channel will be activated to retard energy metabolism to achieve sustaining protection during prolonged ischemia and to decrease the infarct size (IS) (Gysembergh et al., 1998). Chemical metabolites generated from ischemia can also be activators of conditioning. Although ROS can bring severe damage to myocardial cells, a small quantity of ROS can stimulate protective signaling through the oxidation of protective cytosolic kinases (Tullio et al., 2013). Adenosine is a crucial chemical involved in ischemia conditioning. There are 4 kinds of adenosine receptors, A_1_, A_2__A_, A_2__B_, and A_3_, on cardiomyocyte sarcolemma. The A_1_ and A_3_ receptors are essential parts of the ischemic preconditioning (IPC) and their activation function is performed with the combination of K_ATP_ channel (McCully et al., 2001). However, receptors A_2__A_ and A_2__B_ are related to ischemic postconditioning (POC), which should be activated at early stage of ischemia to preserve the protective function (Methner et al., 2010). All the adenosine receptor protective conditioning will be ceased if the receptor signaling such as TNFα is blocked (Lacerda et al., 2009). Remote ischemic conditioning (RIC) can be activated by stromal cell-derived factor-1α (SDF-1α). It has been confirmed that SDF-1α may perform a MI treatment by increasing long-term, stem cell migration, and homing to the heart (Davidson et al., 2013).

## Hypoxia-Related Cardiovascular Diseases

The human body can experience hypoxia under a wide variety of circumstances. We may encounter problems with oxygen extraction due to environmental or physiological reasons. Alternatively, there are pathophysiological circumstances that impair oxygen delivery to the cells, such as respiration system dysfunction, unreached peripheral areas by blood, and occlusion of blood flow by vascular plaques (Thomas and Ashcroft, 2019). In either case, a lack of cellular oxygen has negative consequences for the cardiovascular function which eventually manifests in disease.

A typical example of a hypoxia-related cardiovascular disease is atherosclerosis; a potentially fatal disorder that is characterized by the formation of arterial plaques by the deposition of lipids, macrophages, and other cells in the vessel wall (Weber and Noels, 2011). Endothelial dysfunction is the main cause of atherosclerosis, which is thought to be related to hypoxic conditions. The generation of ROS during hypoxia has a direct effect on the initiation of endothelial dysfunction, especially superoxide (Douglas and Channon, 2014). Superoxide can combine with nitric oxide (NO) and form the harmful product peroxynitrite, which has a damaging effect on lipids and proteins, causing endothelial injuries. As an inflammatory site, endothelial injury can represent a bed for plaque formation (Wang et al., 2012).

Low-density lipoprotein (LDL) is an unstable cholesterol transporter that can easily be oxidized into oxidized LDL (oxLDL). Monocytes migrating to the plaque lesion will differentiate into macrophages and take up oxLDL, thereby turning intofoam cells. Foam cells can exacerbate the inflammatory process and form fatty streak (Insull, 2009). This process can be seen as the initiation of atherosclerosis. Afterward, if chronic hypoxia persists, the fatty streak will generate an atheroma and eventually cause atherosclerosis (Negre-Salvayre et al., 2019).

Another cardiovascular disease that can be induced by hypoxia is pulmonary hypertension. Pulmonary hypertension is defined as mean pulmonary blood pressure sustained over 25mmHg at rest (Humbert et al., 2013). Pulmonary hypertension is induced by hypoxic pulmonary vasoconstriction (HPV) and vascular remodeling (Rimoldi et al., 2012). In a low oxygen environment, HPV is adopted by the lungs as the main strategy to improve oxygen delivery. This response is mechanistically achieved by a rise in calcium influx triggered by ROS signaling. An increase in ROS activates the sarcoplasmic reticulum which leads to the release of Ca^2+^ that enters pulmonary arterial smooth muscle cells and causes vasoconstriction (Siques et al., 2018). This process leads to an increase in pulmonary vasculature blood pressure in order to maintain normal cardiac output, and alongside this, the right ventricle must enhance systolic pressure. The enhanced right ventricular systolic pressure will lead to an increase in both vascular stress and oxygen demand, and a decrease in right coronary artery flow, which is a known compensatory mechanism in pulmonary hypertension (PH) (Oliveira et al., 2020).

The thickness of the pulmonary artery can also be mediated by hypoxic stress. This parameter is normally maintained by the dynamic equilibrium between proliferation and apoptosis of vessel wall cells (Welsh and Peacock, 2013). However, this balance can be interrupted in hypoxic conditions due to the consequent oxidative stress. Oxidative stress can both inhibit the anti-mitogenic factor and lead to the release of mitogenic stimuli (Stenmark and Mecham, 1997). These processes play a vital role in enhancing the proliferation of vessel wall cells, which disturbs the dynamic balance of vessel wall thickness, leading to vascular remodeling (Renna et al., 2013). The blockage of lumen blood flow by hypoxic vascular remodeling is also a contributor to the PH. Moreover, if PH persists, the condition will deteriorate further into another cardiovascular problem, heart failure (Guazzi and Naeije, 2017).

Heart failure is not a single myocardial disease but a progressive disorder where the heart is incapable of filling the left ventricle and/or ejecting sufficient blood to meet the body’s metabolic demands (Kiyuna et al., 2018). Heart failure represents the end-stage of almost all kinds of cardiac diseases, including myocardial infarction, hypertrophy, and hypertension; all of which are associated with high mortality (Garg et al., 2005). According to extensive studies on the pathophysiology of heart failure, mitochondrial abnormalities resulting from hypoxia are a major contributor and a hallmark of heart failure (Okonko and Shah, 2015). The lack of oxygen and subsequent mitochondrial dysfunction causes a sudden decrease in energy supply which can strikingly impair the metabolism of cardiomyocytes. Moreover, the increased generation of ROS can have damaging effects on cell function and integrity that may eventually trigger cardiomyocyte apoptosis. The severe loss of myocardial cells can lead to ventricular remodeling and contractile impairment (Hilfiker-Kleiner et al., 2005). As a consequence, left ventricle dysfunction will lead to decreased cardiac output and may even cause myocardial infarction, which is a critical stage in the progression of heart failure (Struthers, 2005).

## The Roles of Hypoxic HIF-1 Signaling in Cardiovascular Diseases

In response to hypoxia, protective mechanisms will be modulated by activation of the transcriptional factor HIF-1 (Garg et al., 2005). HIF-1 regulates target genes related to inflammation, vascular remodeling, and angiogenensis, which all help the organism respond and adapt to a low oxygen environment. However, these responses are also major contributors to cardiovascular dysfunction (Semenza, 2014a), and once initiated, they can manifest as severe cardiovascular diseases (Abe et al., 2017).

HIF-1 transcriptional factor is a heterodimer consisting of the α-subunit, which includes HIF-1α, HIF-2α, and HIF-3α isoforms, and the HIF-β subunit (Halligan et al., 2016). Under normoxic conditions, the HIF-α subunits are not stabilized and do not modulate their target genes because the subunits are degraded through oxygen-dependent mechanisms (Masoud and Li, 2015). The degradation process starts with hydroxylation of HIF-α. Once the HIF-α is generated, the prolyl hydroxylase domains (PHD) will hydroxylate its highly conserved specific proline residue and form a binding site. Subsequently, the von Hippel-Lindau (VHL) tumor suppressor protein will bind to this binding site, and this reaction makes HIF-α a target for the multiprotein E3 ubiquitin ligase complex to recognize (Semenza Gregg, 2012). Finally, the proteasomal degradation of HIF-α will be induced. However, the hydroxylation process of proline residue by PHD employs oxygen as a reaction substrate, so that the VHL degradation will be inhibited under hypoxic conditions (Figure 1) (Hewitson and Schofield, 2004).

Another HIF degradation process is asparaginyl hydroxylation. This hydroxylase was first identified as a factor inhibiting HIF (FIH). When HIF-α binds to p300-CBP co-activator family, a hydrophobic region will be formed. In this hydrophobic region, an asparagine residue (Asn803 in HIF-α; Asn850 in HIF-2α) is located in the C-terminal of the HIF-α, which is the target of FIH (Kaelin and Ratcliffe, 2008). After the hydroxylation of this asparagine residue, the combination reaction of HIF-α and p300-CBP co-activator will be inhibited. This inhibition will cause the inactivation of HIF transcriptional modulation of downstream target genes (Schodel and Ratcliffe, 2019). However, this hydroxylation is also oxygen-dependent, so that HIF transcription factors can exhibit the regulation effect on target genes.

The beta subunits of HIF are highly conserved and stabilized in the cell nucleus (Metzen, 2003). Under low oxygen conditions, the proteasomal degradation of HIF-α subunits is ceased, the alpha subunits are stabilized and transferred to the nucleus, allowing binding to HIF-β (Liu and Semenza, 2007). When the beta subunits bind to the 1α subunits, the complex forms the HIF-1 transcription factor. Furthermore, it can form HIF-2 when the beta subunits bind to 2α subunits. As the HIF-α/β stabilization is achieved, this transcriptional factor will exhibit its transcriptional function and regulate diverse target genes (Schönenberger and Kovacs, 2015).

Hypoxia-inducible factorHIF transcription factor regulates the expression of target genes by binding to their specific hypoxia response elements (HREs) at the gene loci (Pugh and Ratcliffe, 2003). In response to hypoxia, the protective mechanisms are activated by a network of hundreds of downstream target genes to adapt the body to a low oxygen environment. These responses include a coordinated reduction in oxygen-consuming processes and the promotion of anaerobic metabolic processes (Semenza, 2011). In addition to maintaining ATP balance, the cell must also avoid the overproduction of toxic ROS due to inefficient mitochondrial aerobic respiration (Okamoto et al., 2017). To attenuate the cell-damaging effects of ROS, OXPHOS processes are downregulated, while the anaerobic respiration process, glycolysis, is upregulated (Thomas and Ashcroft, 2019).

As the essential substrate of OXPHOS process, pyruvate must first be reduced to acetyl coenzyme A. This step is catalyzed by pyruvate dehydrogenase, and acetyl coenzyme A is supplied to the TCA cycle (Iyer et al., 1998). A PDH inhibitor, PDK-1, is activated by the HIF-1 transcription under hypoxic circumstances (Wigfield et al., 2008). PDK-1 phosphorylases and inhibits the catalytic domain of PDH. As a result, PDH is driven away from the mitochondria, and the TCA cycle is impaired (Papandreou et al., 2006). In a previous study, HIF-1α null (Hifa^–^/^–^) mouse embryo fibroblasts (MEFs) were unable to activate PDK1, compared to the dramatic increase of PDK1 in isogenic wild-type MEFs (Kim et al., 2006); the decrease of PDH expression is related to HIF-1α but not HIF-2α (Eguchi and Nakayama, 2019).

While the aerobic respiration substrate is reduced, the glycolysis substrates are upregulated by HIF-1 transcription. The expressions of SLC2A1 and SLC2A3 genes are activated by HIF-1 modulation. These genes encode glucose transporters, GLUT1 and GLUT3 (Nishimura et al., 2017). These transporters are upregulated to transfer more glucose to enhance the process of glycolysis. In mouse chondrocytes, GLUT-1 and GLUT-3 expressions were remarkably increased after hypoxic treatment, compared with cells treated in normoxic conditions (Ren et al., 2008). In Figure 1, the increase in the “fast” transporter GLUT-3 can be considered adaptive in hypoxia because this is a more efficient glucose carrier in chondrocytes, compared to other members of this transporter family. Moreover, lactate dehydrogenase A is also upregulated by HIF-1 due to its role in converting pyruvate into lactate (Lee Dong et al., 2015). Hexokinase is the first enzyme to initiate the glycolysis process. This enzyme is encoded by HK-1 and HK-3 gene, which are targets of HIF-1 protective mechanisms as well (Riddle et al., 2000). As the glycolysis process can be carried out smoothly, sufficient ATP will be supplied to maintain normal cell function and metabolism.

During prolonged hypoxia, the generation of ROS becomes a major threat to cell survival (Chi et al., 2010). The overproduction of ROS can trigger several mechanisms that effectively disrupt the proton gradient and lead to the rupture of mitochondrial membrane (Suski et al., 2012). The leakage of mitochondrial content can cause cell death (Bock and Tait, 2019). In order to preserve cell viability, mitochondrial autophagy is activated through the HIF-1 signaling pathway. The selective autophagy regulator, Beclin 1, originally binds to Bcl 2, but when hypoxia occurs, HIF-1 modulation activates BNIP3. The BNIP3 protein has a competitive effect on Beclin 1 on the bond to Bcl 2. The detached Beclin 1 from Bcl 2 triggers selective autophagy (Lu et al., 2018). This autophagy pathway is an adaptive mechanism that protects cell viability during prolonged hypoxic exposure.

HIF-1α, as an important gene modulator to hypoxia, the expression of which has been detected in atherosclerotic lesions from different cell types (Akhtar et al., 2015). Development of atherosclerosis can be promoted by endothelial dysfunction, inflammation, macrophages, and proliferation of smooth muscle cells. Interestingly, all features mentioned above can be induced by HIF regulated pathways (Semenza, 2014b). In Table 1, the contribution of HIF-1α on atherosclerosis has been demonstrated by Shamima’s research (Akhtar et al., 2015). After partial ligation of carotid arteries and high-fat diet (HFD) feeding for over 6 weeks, mice from EC-HIF-1α^–/–^ group and EC-HIF-1α^+/+^group were conducted to atherosclerosis lesion quantification. It was determined that, compared to EC-HIF-1α^+/+^ group mice, the lesion area was reduced in gene silencing group. Endothelial dysfunction and proliferation is a vital feature of atherosclerosis formation, and it has proven to be upregulated by HIF1-α. Cultured endothelial cells were conducted to proliferation measurement, PCNA [proliferating cell nuclear antigen], and Ki67 staining. The level of EC proliferation in HIF-1α^+/+^ group was much higher than that in the HIF-1α gene deletion group (Feng et al., 2017). The accumulation and adhesion of monocytes in endothelial cells also promote the formation of atherosclerosis. The adhesion process can be enhanced depending on upregulated (C–X–C motif) ligand 1 (CXCL1) expression (Zhou et al., 2011). In Shamima’s study, moxLDL and LPA 20:4 were used to induce monocyte adhesion in mouse aortic EC (MAECs). As the monocyte adhesion cannot be induced in the HIF-1α silencing group, it is indicated that the adhesion-depending CXCL-1 expression is regulated by HIF-1α (Table 1) (Akhtar et al., 2015).

**TABLE 1:** Experimental models used, the protocols, and the role exercised by HIF in studies involved in the article.

Model of the study	Methods	Outcome and experimental evidence	References
Mouse model with high-fat diet induced atherosclerosis	The lesion formation of EC-Hif1a-/- mice and wild type were determined after partial carotid ligation and HFD feeding.	EC-Hif1a-/- mice generated reduced lesion area compared to EC-Hif1a + / + mice.	Akhtar et al., 2015
Mouse aortae endothelial cell model after HFD feeding	Monocyte adhesion to MAECs was determined after the stimulation of LPA20:4, LPA18:0, moxLDL or nLDL.	The moxLDL-and LPA20:4-induced monocyte adhesion was abolished by silencing Hif-1a in MAECs. Upregulation of HIF1a helps MoxLDL-derived unsaturated LPAs promote CXCL1-dependent monocytes adhesion.	Akhtar et al., 2015
Mouse aortae endothelial cell model after HFD feeding	Endothelial cell proliferation was measured between Hif-1a silencing group and the control group.	The proliferation of ECs was decreased in gene silencing group, demonstrating that EC proliferation is regulated by Hif-1a.	Feng et al., 2017
Mouse pulmonary hypertension model induced by hypoxia-induced mitogenic factor (HIMF) injection	The level of medial thickening was determined between HIF-1α^+/+^ and HIF-1α^±^ group after HIMF injection.	An obvious medial thickening was induced by HIMF in the HIF-1α^+/+^ group, while the process was dismissedin the HIF-1α^±^ group, indicating the regulation effect of HIF-1α in the PH development.	Johns et al., 2016
Mouse model by normoxia or chronic hypoxia (10% O_2_) for 30 days	HIF-1α deletion was performed in 2 ways on model group mice, followed by normoxia and chronic hypoxia exposure to all groups.	HIF-1α-SMM-Cre mice generated decreased arterial wall thickness, while the control group mice developed significant vascular remodeling in arteries, showing HIF-1α as an important part in vascular remodeling regulation.	Ball et al., 2014

During the development of pulmonary hypertension and heart failure, HIF-1α-induced vascular remodeling also plays an important role (Schultz et al., 2006). Hypoxia-induced mitogenic factor (HIMF) regulation is a crucial part of the EC proliferation phase in pulmonary hypertension. Johns’ group used HIMF to induced medial thickness on mouse model, to compare the level of EC proliferation between HIF-1α ± group and wild type (WT) group. The result turned out that the vessel wall thickness process was abolished in the HIF-1α ± group compared to the WT group. This outcome indicated that HIF-1α is a vital downstream regulator in the process of HIMF-induced pulmonary hypertension, which means HIF-1α plays an important part in PH development (Johns et al., 2016). Ball’s group performed two ways, homozygous conditional deletion of HIF-1 a combined with tamoxifen-inducible smooth muscle-specific Cre recombinase expression, to achieve the gene silencing of HIF-1α. Both the HIF-1α-SMM-Cre group and WT group mice were exposed to either normoxia or chronic hypoxia (CH, 10% O_2_). In normoxic situation, both groups exhibited no vascular remodeling, while in CH situation, the obvious vessel wall thickness induced in WT group was significantly reduced in gene negative group (Table 1) (Ball et al., 2014).

According to the researches above, HIF-1α is closely correlated with the developing process of several cardiovascular diseases, such as endothelial dysfunction, smooth muscle proliferation, inflammation, and angiogenesis through the transcription of vascular endothelial growth factor (VEGF), erythropoietin (EPO), CXCL1, etc (Figure 1). The important role of HIF-1α in CVD development makes it a convincing target of cardioprotective treatment.

## Hypoxia-Inducible Factors and Potential Therapeutic Targets

A lack of blood flow (ischemia) underlies numerous cardiovascular, including myocardial infarction and atherosclerosis. Furthermore, ischemia not only underlies cardiovascular diseases but also diabetes and other chronic diseases (Howell and Tennant, 2014). To treat the ischemic myocardium in myocardial infarction, the standard therapy is to reperfuse the ischemic area. This standard treatment may paradoxically cause additional injury to the myocardium, which is termed ischemic-reperfusion injury (IRI) (Martin-Puig et al., 2015). IRI can trigger a sudden increase in ROS and intracellular calcium overload at the reperfusion site, which can eventually cause mitochondrial dysfunction and even cell death. To protect the cell from this cascade of events, researchers have adopted the process of ischemic preconditioning, whereby the cell is subjected to short cycles of ischemia-reperfusion before the longer, chronic phase of IRI. Due to its short period, this process is not lethal, and it is known to adapt and protect the myocardium to the later damaging effects of IRI. Because of the differences in the partial pressure of oxygen between the ischemic and reperfusion periods, it can be hypothesized that the HIF signaling pathway may participate in the mechanisms underlying ischemic preconditioning.

Previous studies have demonstrated that HIF transcription factor and its downstream target genes have cardioprotective abilities through the modulation of mitochondrial metabolism, cell function, and angiogenesis. All these reactions adjust cellular function in the hypoxic area to the low oxygen environment and maintain normal cell homeostasis, which is vital for the human body when facing hypoxic stress. Therefore, the HIF pathway provides a new target to develop strategies for treating ischemic diseases and reducing reperfusion injury.

As a promoter of atherosclerosis development, HIF may not be considered for therapeutic treatment of this disease. While protective in the short term, the angiogenesis induced by HIF-1 transcription that forms collateral vessels may result in terrible consequences in atherosclerosis patients (Jain et al., 2018). However, this conclusion is based on current research results, which are mostly focusing on the HIF-1 signaling pathway, particularly the HIF-α subunits. More study is needed on other members of the hypoxia-inducible factor family and the HIF-β subunits. The physiological mechanisms behind the modulation of these transcription factors may possess great potential for treating cardiovascular diseases such as atherosclerosis.

Pulmonary hypertension is another cardiovascular disease associated with HIF modulation. The proliferation of pulmonary arterial smooth muscle cells (PASMCs) and endothelial cells (ECs) which are activated by HIF-1 and HIF-2 transcription, respectively, contributes toward the increase in pulmonary blood pressure (Ahmad et al., 2013). Strategies that target the inhibition of HIF transcription in PH patients could be an interesting new perspective in the treatment of hypertension. For example, it has been demonstrated that HIF-2 inhibited by C76 is capable of relieving pulmonary artery blood pressure in different models (Dai et al., 2018).

In summary, according to current researchers, the complicated mechanisms of HIF transcription have yet be completely revealed. Further research is necessary in order to obtain a complete therapeutic picture of HIF in the treatment of cardiovascular diseases (Burgueno et al., 2013).

## Author Contributions

ML and GG contributed to the drafting and revision of the manuscript. YW and ZW contributed to the critical revision of the manuscript. QF contributed to the substantial contributions to conception and design.WXandXWcontributed to the critical revision of the manuscript and final approval of the version for submission. All authors contributed to the article and approved the submitted version.

## Conflict of Interest

The authors declare that the research was conducted in the absence of any commercial or financial relationships that could be construed as a potential conflict of interest.
